# A gamified choice experiment of traditional African vegetable varieties in West Africa

**DOI:** 10.1371/journal.pone.0345915

**Published:** 2026-03-25

**Authors:** Sidol Houngbo, Simon Codjo, Mathieu A. T. Ayenan, Rodrigue S. Kaki, Benoît Govoeyi, Modeste Dohou, Christelle Komlan, Irene M. Mitchodigni, N'Danikou Sognigbe, Maarten van Zonneveld, Pepijn Schreinemachers

**Affiliations:** 1 World Vegetable Center, West and Central Africa (WCA)–Coastal and Humid Regions, Cotonou, Benin; 2 World Vegetable Center, East and Southern Africa, Arusha, Tanzania; 3 World Vegetable Center, Shanhua, Taiwan; 4 World Vegetable Center, East and Southeast Asia, Bangkok, Thailand; Lusofona University of Humanities and Technologies: Universidade Lusofona de Humanidades e Tecnologias, PORTUGAL

## Abstract

Understanding trait preferences of value chain actors is essential for designing demand-driven breeding programs for traditional African vegetables (TAVs). The objective of this study was to assess the varietal preferences of farmers, traders, and consumers for amaranth, okra, and jute mallow in Benin and Mali. We employed a gamified choice experiment, following the Bradley–Terry model and recursive partitioning, to identify preference patterns and segment participants by country, age, and gender. Different preference patterns appeared across segments, showing clear social and geographic diversity in varietal needs. Traders and consumers consistently focused on market and organoleptic traits, specifically, leaf integrity and taste for amaranth, high mucilage content (viscosity) for okra, and both leaf integrity and viscosity for jute mallow. Farmers prioritized a range of agronomic traits. For okra, farmers specifically valued harvesting duration, disease resistance, and fruit yield per plant, reflecting the crop’s dual role in income generation and nutritional security. Farmers in Mali placed greater emphasis on drought tolerance across all three crops than farmers in Benin. The findings highlight the need for TAV breeding and variety introduction programs to consider not only agronomic performance but also market preferences and organoleptic qualities.

## 1. Introduction

Malnutrition continues to pose a significant global health challenge and remains a primary cause of mortality [1]. Malnutrition affects approximately one in three preschool-aged children in developing countries [2]. The issue is particularly severe in sub-Saharan Africa (SSA), where pervasive undernutrition coexists with increasing rates of overweight, diet-related non-communicable diseases, and micronutrient deficiencies. In 2022, the prevalence rates among children under five in this region were estimated to be 31.3% (59.4 million) for stunting, 5.7% (10.3 million) for wasting, and 3.7% (6.6 million) for obesity [3].

In Africa, the share of people unable to afford a healthy diet rose from 64.1 percent in 2019 to 66.6 percent in 2024, corresponding to an increase from 864 million to 1 billion [4]. A key driver of malnutrition is the low consumption of nutrient-rich foods, including vegetables. Insufficient vegetable intake is widely recognized as a contributor to poor health and early mortality [5]. SSA has the lowest per capita vegetable intakes worldwide, averaging less than 100 g per day—well below the World Health Organization’s recommended minimum of 400 g daily [5]. Vegetables are an indispensable part of healthy diets and are crucial for food and nutrition security [6]. SSA has a diversity of traditional vegetables that could help improve nutritional security, but these have been understudied [7].

Towns and Shackleton [8] described traditional African vegetables (TAVs) as “plant species that are native or naturalized to Africa, well adapted to or chosen for local conditions, with parts consumed as vegetables”. Their cultivation, gathering, preparation, and consumption are deeply rooted in local cuisine, culture, folklore, and language. Many TAVs are rich in micronutrients and health-promoting phytochemicals [7,9]. They are also easy to add to farming systems as they need little space, fit into short crop rotations, and many are identified as climate-resilient [6,10].

Investments in TAV variety improvement and seed system development have been minimal [11]. As a result, farmers depend on informal seed sources that supply planting material of inconsistent quality [12]. Limited investment has also meant that TAVs have not benefited from formal-sector plant breeding [7]. Enhancing variety adoption and breeding based on the trait preferences of value chain actors—including farmers, traders, and consumers—is vital to meet diverse expectations. Studying roots, tubers and bananas, Thiele et al. [13] showed that neglecting consumer-preferred traits in breeding programs has restricted the adoption of improved varieties and slowed varietal turnover. However, studies on consumer trait preferences for TAVs are rare, as most prior research focused only on farmers [11,14–18].

Mitchodigni et al. [19] studied TAV trait preferences among farmers, traders, and consumers in West Africa to inform priority setting in breeding. They relied on qualitative data and did not consider differences in preferences within groups. This study measures trait preferences for TAVs among vegetable value chain actors through a choice game experiment, offering more detailed information to help develop and promote preferred TAV varieties. The objectives are to (i) identify trait preferences for TAVs among farmers, traders, and consumers; (ii) examine heterogeneity in preferences within these groups by analyzing various sociodemographic combinations; and (iii) detect mismatches in preferred traits across the value chain. We focus on three TAVs: amaranth (*Amaranthus* spp.), okra (*Abelmoschus esculentus*), and jute mallow (*Corchorus olitorius*). These crops are popular in West Africa, have been identified as opportunity crops, and have the potential to become economically important crops [20]. However, access to quality seed for these crops remains a challenge, and there is only limited choice of improved varieties in local markets [19,21,22]. The insights from this research aim to provide breeders with more precise data and support the development of TAV value chains.

Stated-choice experiments are among the most widely used participatory methods for prioritizing varietal traits [23–27]. Such experiments provide detailed data on preferences across a diverse range of respondents. However, a major drawback is that stated-choice experiments often impose a high cognitive load, which can affect the results [28]. Respondents are asked to evaluate and compare multiple complex profiles, each including all traits under study. This makes the task mentally demanding, especially when the number of traits and levels is large, which can lead to overlooking certain traits. To reduce cognitive effort, this study uses a gamified choice experiment that simplifies decision-making by replacing multi-attribute profile comparisons with successive pairwise trait comparisons. This method is suitable for low-literacy contexts because respondents compare only two traits at a time, reducing complexity and cognitive load. It makes the tasks more engaging while still providing detailed and reliable results [29].

The rest of this paper is organized as follows: The second section provides more details on the methodology. The third section discusses the main findings. The fourth section interprets the results, and the fifth outlines the conclusions and implications for intervention.

## 2. Materials and methods

### 2.1. Study area

This study was conducted in Benin and Mali, two West African countries facing persistently low per capita vegetable consumption and significant health issues related to the triple burden of malnutrition [30]. The research focused on these countries’ main production areas of amaranth, okra, and jute mallow, chosen based on recommendations from local agricultural extension officers. In Benin, the study sites included the southern districts of Athieme (rural) and Abomey-Calavi (urban), as well as the northern districts of Parakou (urban) and Natitingou (periurban). In Mali, the research took place in Bamako (urban) and Koulikoro (urban).

### 2.2. Sampling

To determine the required sample size for farmers and traders in each district, we followed the rule of thumb for discrete choice experiments proposed by Orme [31]. This rule suggests that the number of respondents (*n*) should satisfy the following condition [32]:


$$n\geq \text{500}\cdot \frac{L^{max}}{J\cdot S}$$ (1)

where $L^{max}$ is the largest number of levels of any attribute, *J* is the number of alternatives per choice task, and *S* is the number of choice sets each respondent completes. Based on this rule and consistent with previous behavioral studies [33], at least 30 farmers and 30 traders were randomly selected in each district to participate in our choice game experiment. In practice, we first compiled a comprehensive, though not exhaustive, list of farmers and traders, from which participants were randomly selected for the choice game experiment. The farmers’ list was compiled with the assistance of local agricultural extension officers, while the traders’ list was based on a preliminary informal survey conducted in the largest vegetable market of each urban district. The sample size was increased in some districts to enhance the reliability of the data analysis. The final sample included 104 farmers and 60 traders in Benin and 85 farmers and 67 traders in Mali (Table 1).

**Table 1:** Sample distribution.

Country	District	Value chain actors		
		Farmers	Traders	Consumers
Benin	Abomey-Calavi	20	30	280
	Athieme	24	–	–
	Parakou	39	30	329
	Natitingou	21	–	–
	**Total**	104	60	609
Mali	Bamako	55	35	384
	Koulikoro	30	32	343
	**Total**	85	67	727

We determined the consumer sample size based on the total population of each selected district. The calculation followed Kothari’s [34] formula:


$$n=\frac{z^{2}Np(1-p)}{e^{2}(N-1)+z^{2}p(1-p)}$$ (2)

where *n* is the required sample size, *z* is the critical value at the desired confidence level (1.96 for a 95% confidence level), *N* is the size of the target population, *e* is the margin of error (set at 5%), and *p* is the estimated population proportion with the characteristic of interest. The estimated minimum sample sizes ranged from 278 to 280 across districts. In total, 609 consumers in Benin and 727 consumers in Mali were sampled to participate in our experiment (Table 1). Consumers were randomly approached in the vegetable sections of the largest markets in each urban district, informed about the study objectives, and invited to participate in the experiment after providing consent.

It is noted that farmers were sampled from all study districts, whereas traders and consumers were selected only from urban districts that host the largest markets for amaranth, okra, and jute mallow in the study countries.

### 2.3. Experimental design

Our choice game experiment is inspired by Steinke and van Etten [29]. It uses pairwise choice sets involving two traits per choice situation, with each trait presented at two levels (“high” and “low”), to reduce participants’ cognitive load during decision-making. The selection of traits and the determination of locally appropriate high and low trait levels were guided by exploratory investigations by Mitchodigni et al. [19], which identified the most important attributes for farmers, traders, and consumers. These investigations were conducted in the same countries, districts, and on the same crops as the current study. They included 12 focus group discussions with vegetable farmers (five to nine participants each), 28 interviews with traders, and 34 interviews with consumers.

**Fig 1:**
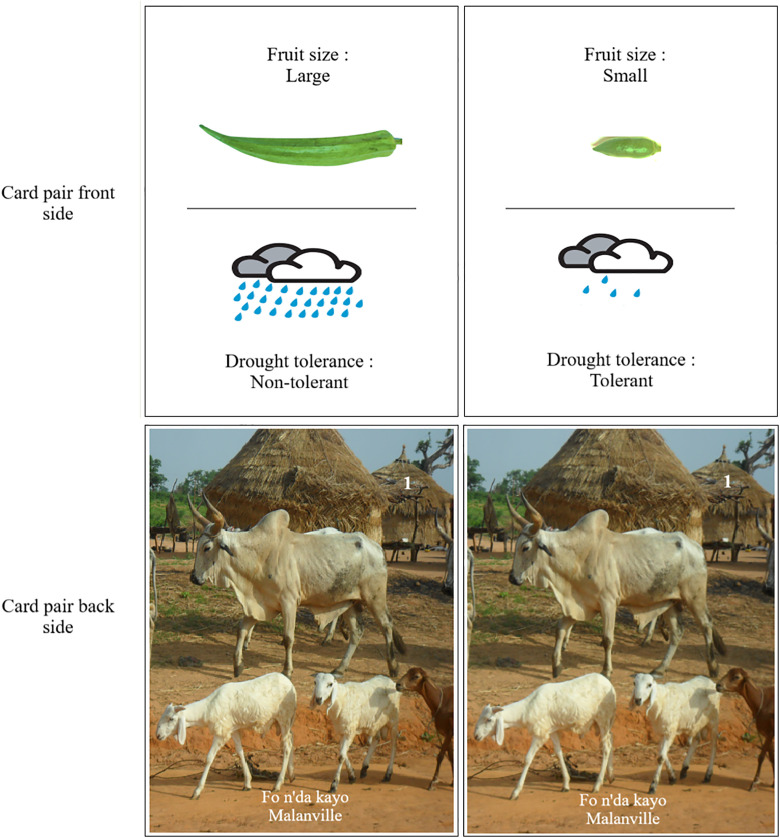
Example of front and back sides of a pair of cards used in the okra trait choice game for farmers in Benin. Photo credit: The authors.

Tables 2 and 3 provide details on the traits and trait levels used to design the experiment. For farmers, the choice game included seven traits per crop, resulting in $\left(\frac{7}{2}\right)$ = 21 pairwise choices per crop. For traders and consumers, the design included five traits for amaranth and jute mallow (10 pairwise choices each) and six traits for okra (15 pairwise choices). Different varietal traits were used for farmers and consumers/traders, as preliminary investigations suggested that the two groups have distinct priorities and decision-making criteria [19]. In each pairwise choice, respondents indicated their preference between two hypothetical scenarios, each defined by the same two traits, with the trait levels presenting a trade-off. For example, one choice was between “*small size* but *tolerant to drought*” and “*large size* but *non-drought-tolerant*” (Fig 1). Participants were required to choose their preferred option in all pairwise comparisons by evaluating the trade-off between the two traits. We then converted the choices into wins and losses for each trait. In the example above, if the participant chose the first option, *drought tolerance* would score a win over *large size*. We used this procedure to design the experiment and collect data for each crop and actor.

**Table 2:** Traits and trait levels used to design the choice experiment for farmers.

Crops	Traits	Low level	High level
Amaranth	Color	Red	Green
	Leaf size	Small	Large
	Multiple harvest	Do not regenerate	Regenerate
	Flowering	Early	Late
	Branching	Unbranched	Ramified
	Disease resistance	Non-resistant	Resistant
	Drought tolerance	Non-tolerant	Tolerant
Okra	Color	Light green	Dark green
	Size	Short/ small	Long
	Time to the first fructification	Long/ late	Short/ early
	Harvesting duration	Short	Long (2–3 months)
	Number of fruits per plant	Few/Low	Many/ High
	Disease resistance	Non-resistant	Resistant
	Drought tolerance	Non-tolerant	Tolerant
Jute mallow	Color	Light green	Dark green
	Leaf size	Small	Large
	Regeneration ability	Do not regenerate	Regenerate
	Flowering	Early	Late
	Branching	Unbranched	Ramified
	Disease resistance	Non-resistant	Resistant
	Tolerance to water stress	Non-tolerant	Tolerant

**Table 3:** Traits and trait levels used to design the experiment for traders and consumers.

Crops	Traits	Low level	High level
Amaranth	Size	Small	Large
	Freshness	Wilted	Fresh
	Color	Red	Green
	Taste	Bland	Succulent
	Physical integrity of leaves	Holey/ with holes	No holes
Okra	Size	Small	Large
	Freshness	Wilted	Fresh
	Color	Light green	Dark green
	Hardness	Hard	Not hard
	Texture	Not smooth	Smooth
	Viscosity	No slime	Slime
Jute mallow	Size	Small	Large
	Freshness	Wilted	Fresh
	Color	Light green	Dark green
	Viscosity	No slime	Slime
	Physical integrity of leaves	Perforated	No holes

Our choice game was designed to mimic the structure of card and lottery games, which are popular in West Africa and elsewhere. To implement the game, we created simple pictograms to represent traits and their levels in hypothetical varieties. Each variety was shown as a playing card. For farmers, we made 21 pairs of cards per crop (42 in total per crop). For traders and consumers, we made 10 pairs of cards for amaranth and jute mallow (20 cards each) and 15 pairs for okra (30 cards). Each pair of cards had hypothetical varieties on the front and a common image on the back, making them easy for respondents to recognize. The images on the back side showed different municipal sites in Benin and Mali. These sites were picked because they were distinctive, helping participants recognize the card pairs quickly. An example of a card pair is shown in Fig 1.

The gameplay and data collection followed seven steps. First, participants spread out a full set of cards randomly and face-down on a table. Multiple players were allowed to play the choice game simultaneously. Second, the enumerator drew a municipal item, a small picture representing a municipal site, and announced it; each item corresponded to an image on the back of the player cards. Third, each player searched for the two cards containing the announced item within their set and flipped them over. Fourth, the enumerator explained the trade-off represented by the front sides of the flipped card pair and asked the player to choose one of the two hypothetical varieties. Fifth, each player selected one card and raised it toward the enumerator, without showing it to the other players or discussing their choice. No passes or ties were allowed. The pair of cards was then set aside. Sixth, the enumerator recorded the players’ choices and repeated the process until no cards remained on the table. Before the gameplay began, participants were informed about this procedure. They were also asked to complete a consent form before the gameplay. This form collected participants’ sociodemographic characteristics.

### 2.4. Data collection

Data collection took place in July and August 2022. The choice game experiment was conducted several times, with sessions involving either individual participants or groups of five to nine participants, chosen randomly. Overall, we recorded the choices of 189 farmers, 127 traders, and 1,336 consumers. We also gathered data on participants’ sociodemographic characteristics using a digitized form, which may serve as potential covariates in the trait prioritization analysis.

### 2.5. Data analysis

We applied the Bradley–Terry model to the gameplay data to estimate the relative importance of the different traits included in the experiment [35]. The model defines the probability that trait $T_{j}$ is preferred over trait $T_{k}$ in a paired comparison [36]. This probability is given by:


$$P\left(\pi_{j},\;\pi_{k}\right)=\frac{\pi_{j}}{\pi_{j}+\pi_{k}}$$ (3)

where $\pi_{j}$ and $\pi_{k}$ are positive parameters representing the positions of traits on the preference scale. In other words, each trait is assigned a positive worth parameter (π>0). The logit form of the model can be expressed as follows:


$$P\left(\beta_{j},\;\beta_{k}\right)=\frac{exp(\beta_{j}-\;\beta_{k})}{1+exp(\beta_{j}-\;\beta_{k})}$$ (4)

where β are the parameters to be estimated. The relative importance of each trait is obtained by calculating a normalized worth utility ($W_{j}$), which is a probability-like measure of importance relative to a reference trait.


$$W_{j}=\frac{\pi_{j}}{\sum_{k}\pi_{k}}$$ (5)

The Bradley–Terry model can be combined with “recursive partitioning” to segment participants into subgroups with similar preferences. In this approach, participant characteristics serve as “splitting variables” to define the groups, allowing the model to account for heterogeneity in preferences. For instance, the model can test whether participants from Benin and Mali exhibit different preferences by using country as a splitting variable. If the difference is statistically significant, the participants are divided into two groups corresponding to each country. This method works with both categorical and continuous variables. For continuous variables, such as age, the model searches for the split point that maximizes model improvement. If a significant difference is detected, participants are separated into two subgroups: one above the split point (e.g., older than 25 years) and one below (e.g., younger than 25 years). The procedure is recursive: after an initial split, the same analysis is applied to each resulting subgroup, continuing until no further significant differences are found. The final output is a regression tree structure consisting of binary splits, representing participant subgroups with homogeneous preference patterns.

For this study, we fitted two types of Bradley-Terry models to the data: one without any splitting variables and one incorporating recursive partitioning with four splitting variables (country, gender, age, and household size), without interaction effects and under the assumption of trait independence. We, thus, fitted two models for each crop and each value chain actor. The models were estimated using the “*BradleyTerry2*” [37] and “*psychotree*” [38] packages in R software (version 4.4.2) [39].

### 2.6. Ethical considerations

The study plan was reviewed and approved by the Institutional Biosafety and Research Ethics Committee (IBREC) of the World Vegetable Center (Registration No. 2022−014, dated 13 June 2022). The participants provided written informed consent to participate in this study.

## 3. Results

### 3.1. Farmers’ trait preferences

The Bradley–Terry model results for amaranth showed that farmers valued all traits significantly, with drought tolerance used as the reference (Table 4). Resistance to diseases, color, multiple harvests (or regenerative ability), and branching had significantly higher worth than drought tolerance, while flowering and leaf size had significantly lower worth. Overall, disease resistance, color, and multiple harvests were the most preferred traits among farmers, together accounting for 65.8% of the total value.

**Table 4:** Bradley–Terry model results for farmers’ pairwise gameplay choices, without recursive partitioning (N = 189).

Crops	Traits	Normalized worth estimates	Standard error	*Z* value	p(>|z|)	Log- Likelihood
Amaranth	Color	0.228	0.084	9.210	<0.001***	−93.828
	Leaf size	0.068	0.083	−5.241	<0.001***	
	Multiple harvests	0.186	0.083	6.883	<0.001***	
	Flowering	0.041	0.088	−10.746	<0.001***	
	Branching	0.127	0.081	2.273	0.023*	
	Disease resistance	0.244	0.084	9.953	<0.001***	
	Drought tolerance	0.105				
Okra	Color	0.061	0.085	−6.657	<0.001***	−82.045
	Size	0.029	0.093	−14.092	<0.001***	
	Time to the first fructification	0.007	0.123	−21.832	<0.001***	
	Harvest duration	0.298	0.088	11.542	<0.001***	
	Number of fruits per plant	0.232	0.086	8.925	<0.001***	
	Disease resistance	0.263	0.087	10.262	<0.001***	
	Drought tolerance	0.108				
Jute mallow	Color	0.112	0.093	5.012	<0.001***	−86.411
	Leaf size	0.077	0.093	0.837	0.403	
	Regeneration ability	0.179	0.095	9.737	<0.001***	
	Flowering	0.041	0.098	−5.706	<0.001***	
	Branching	0.227	0.097	11.987	<0.001***	
	Disease resistance	0.291	0.099	14.125	<0.001***	
	Tolerance to water stress	0.071				

*p < 0.05, **p < 0.01, ***p < 0.001. *Drought tolerance* is the reference trait in the amaranth and okra models; *tolerance to water stress* is the reference trait in the jute mallow model.

For okra, the Bradley–Terry model results showed that farmers valued traits such as harvesting duration, disease resistance, and the number of fruits per plant more highly than drought tolerance (which was used as the reference trait in the model). Conversely, color, size, and time to first fructification were valued significantly less than drought tolerance. Collectively, harvest duration, disease resistance, and the number of fruits per plant accounted for 79.3% of the total worth, making them the most important traits farmers preferred in okra varieties.

The results for jute mallow showed that, compared to tolerance to water stress, all traits except leaf size were significantly preferred by farmers. Farmers strongly preferred disease resistance, branching, regenerative ability, and color over water stress tolerance, while flowering was considered less valuable. Overall, they prioritized disease resistance, branching, and regenerative ability, which together made up 69.7% of their preferences for jute mallow varieties.

**Fig 2:**
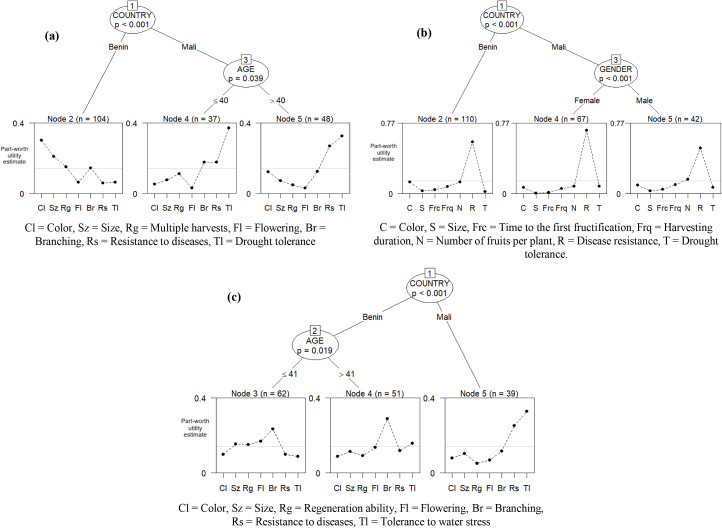
Bradley–Terry model trees showing the recursive partitioning of farmers’ preferences for traits of amaranth (a), okra (b), and jute mallow (c) (N = 189).

Fig 2 shows Bradley–Terry model trees of farmers’ trait preferences obtained through recursive partitioning for amaranth (a), okra (b), and jute mallow (c). Additional details on the recursive partitioning model results are provided in S1–S6 Tables. For amaranth, the model divided the farmer sample into three significant subgroups based on country and age: farmers from Benin; farmers from Mali aged 40 or younger; and farmers from Mali over 40. Gender and household size did not lead to splits for amaranth. These three farmer groups showed different trait priorities. Farmers in Benin valued color, leaf size, multiple harvests, and branching more than drought tolerance. These traits made up about 81% of their variety preference (see S1 Table). Farmers in Mali aged 40 or younger preferred drought tolerance, followed by multiple harvests. Together, these accounted for 48.4% of their total worth. Farmers in Mali aged over 40 prioritized drought tolerance and branching, which together accounted for 45.3% of their variety preferences.

For okra, the model divided the farmer sample into three main groups by country and gender: farmers in Benin, female farmers in Mali, and male farmers in Mali (Fig 2). Beninese farmers prioritized disease resistance over drought tolerance, with disease resistance alone making up 56.7% of their variety preference (see S2 Table). They also valued the number of fruits per plant and color significantly more than drought tolerance. Female farmers in Mali valued disease resistance more than drought tolerance, with disease resistance alone accounting for 69.3% of their variety preference (S2 Table). Male farmers in Mali also preferred disease resistance, followed by the number of fruits per plant, over drought tolerance. These traits together represented 65.9% of their preferences (S2 Table).

The model estimated for jute mallow also identified three key farmer subgroups by country and age: farmers in Benin aged 41 or younger, farmers in Benin older than 41, and farmers in Mali (Fig 2). No additional splits were observed based on gender or household size. Farmers in Benin aged 41 or younger prioritized branching, flowering, regeneration ability, and leaf size over drought tolerance. These traits accounted for 71.2% of their variety preferences (see S3 Table). Farmers in Benin older than 41 highly valued branching, which they considered most important, and drought tolerance over all other traits. Farmers in Mali placed the highest importance on drought tolerance, which alone made up 32.9% of their preference (S3 Table).

### 3.2. Traders’ trait preferences

Table 5 shows the results of the Bradley–Terry models for amaranth, okra, and jute mallow as estimated for traders. For amaranth, traders valued the physical integrity of the leaves most, which alone accounted for 33.4% of their preference across amaranth varieties. In comparison, they rated leaf size, freshness, and color less than the physical integrity of the leaves. For okra, traders prioritized viscosity (high mucilage content) over all other traits, placing less importance on color, texture, size, and hardness. For jute mallow, the physical integrity of the leaves was the most valued, followed by viscosity. These two traits together made up 72.5% of their preferences. Meanwhile, freshness, color, and size were rated much lower in importance. The Bradley–Terry model with recursive partitioning produced only one node for amaranth, okra, and jute mallow, indicating no significant subgroup structure among traders.

**Table 5:** Bradley–Terry model results for traders’ pairwise gameplay choices, without recursive partitioning (N = 127).

Crops	Traits	Normalized worth estimates	Standard error	*Z* value	p(>|z|)	Log- Likelihood
Amaranth	Size	0.042	0.189	−10.981	<0.001***	−74.305
	Freshness	0.139	0.159	−5.495	<0.001***	
	Color	0.233	0.156	−2.312	0.021*	
	Taste	0.253	0.156	−1.783	0.075	
	Physical integrity of leaves	0.334				
Okra	Size	0.031	0.166	−14.333	<0.001***	−52.609
	Freshness	0.453	0.151	1.934	0.053	
	Color	0.104	0.149	−7.918	<0.001***	
	Hardness	0.011	0.194	−17.844	<0.001***	
	Texture	0.062	0.155	−10.946	<0.001***	
	Viscosity	0.339				
Jute mallow	Size	0.045	0.175	−12.850	<0.001***	−61.442
	Freshness	0.147	0.154	−6.879	<0.001***	
	Color	0.084	0.161	−10.054	<0.001***	
	Viscosity	0.302	0.151	−2.235	0.025*	
	Physical integrity of leaves	0.423				

*p < 0.05, **p < 0.01, ***p < 0.001. *Physical integrity of leaves* is the reference trait in the amaranth and jute mallow models; *viscosity* is the reference in the okra model.

### 3.3. Consumers’ trait preferences

Overall, consumer preferences were similar to those of traders (Table 6). For amaranth, they prioritized the physical integrity of leaves, followed by taste. Together, these traits made up 64.1% of consumer preferences. For okra, viscosity was considered the most valuable trait, aligning with trader preferences. For jute mallow, consumers placed higher value on the physical integrity of leaves than on viscosity. These two traits accounted for 75.7% of their variety preferences.

**Table 6:** Bradley–Terry model results for consumers’ pairwise gameplay choices, without recursive partitioning (N = 1336).

Crops	Traits	Normalized worth estimates	Standard error	*Z* value	p(>|z|)	Log-Likelihood
Amaranth	Size	0.046	0.045	−45.96	<0.001***	−587.119
	Freshness	0.137	0.039	−24.97	<0.001***	
	Color	0.176	0.038	−18.71	<0.001***	
	Taste	0.282	0.038	−6.422	<0.001***	
	Physical integrity of leaves	0.359				
Okra	Size	0.035	0.043	−54.881	<0.001***	−257.584
	Freshness	0.383	0.039	0.792	0.428	
	Color	0.088	0.039	−36.475	<0.001***	
	Hardness	0.017	0.048	−64.975	<0.001***	
	Texture	0.106	0.039	−32.156	<0.001***	
	Viscosity	0.371				
Jute mallow	Size	0.038	0.045	−52.358	<0.001***	−494.372
	Freshness	0.081	0.039	−29.768	<0.001***	
	Color	0.125	0.041	−39.320	<0.001***	
	Viscosity	0.356	0.038	−3.051	0.002**	
	Physical integrity of leaves	0.401				

*p < 0.05, **p < 0.01, ***p < 0.001. *Physical integrity of leaves* is the reference trait in the amaranth and jute mallow models; *viscosity* is the reference in the okra model.

**Fig 3:**
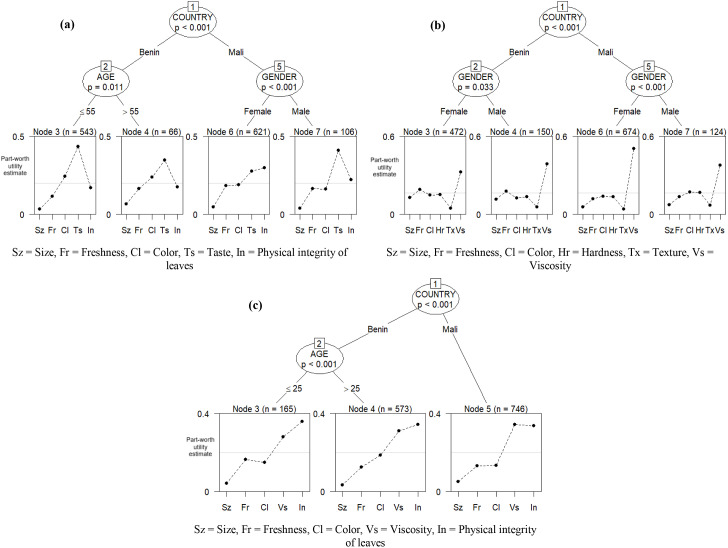
Bradley–Terry model trees showing the recursive partitioning of consumers’ preferences for traits of amaranth (a), okra (b), and jute mallow (c) (N = 1336).

Unlike traders, the Bradley–Terry model for consumers identified subgroup structures. Specifically, it divided the consumer sample into four subgroups for amaranth (based on country, age, and gender), four subgroups for okra (based on country and gender), and three subgroups for jute mallow (based on country and age). For amaranth, the significant consumer segments were: consumers in Benin aged 55 or younger, consumers in Benin aged 55 or older, female consumers in Mali, and male consumers in Mali (Fig 3). Consumers in Benin aged 55 or younger prioritized taste and color over the physical integrity of leaves (the reference trait in the model). Together, these traits accounted for 67.9% of their variety preference (S4 Table). Consumers in Benin older than 55 placed significantly more value on taste than on the physical integrity of the leaves. Female consumers in Mali placed the highest value on the physical integrity of leaves among all other traits. Male consumers in Mali valued taste and the physical integrity of leaves most, which together made up 63.3% of their preference for amaranth (S4 Table).

The model for okra also divides the consumer sample into four subgroups: female consumers in Benin, male consumers in Benin, female consumers in Mali, and male consumers in Mali (Fig 3). All of these subgroups significantly valued the traits considered at different levels but prioritized okra viscosity above all other traits. Viscosity alone accounted for 32.7–50.7% of consumers’ preferences for okra varieties (see S5 Table).

For jute mallow, the model identified three key consumer groups: consumers in Benin aged 25 or younger, consumers in Benin older than 25, and consumers in Mali (Fig 3). Although these groups valued the traits differently, all gave the highest priority to the physical integrity of the leaves over other traits. This trait alone made up 33.8% to 35.9% of their preferences for jute mallow varieties (S6 Table). Besides physical integrity of the leaves, consumers in Benin aged 25 or younger mostly prioritized viscosity second, while consumers in Benin older than 25 and those in Mali primarily prioritized color as their second choice.

## 4. Discussion

This study measured farmers’, traders’, and consumers’ preferences for amaranth, okra, and jute mallow using a choice game experimental design. We identified key segments of farmers and consumers based on country, age, and gender. These insights are useful for breeders and seed companies, as they support demand-driven breeding and the development of a seed system for TAVs [21].

When comparing trait preferences across actors within TAV value chains, traders and consumers consistently prioritize organoleptic traits such as leaf integrity and taste for amaranth, viscosity for okra, and both leaf integrity and viscosity for jute mallow. In contrast, farmers focus on a broader range of traits, and varietal preferences vary across farmer segments. It is worth noting that a trait like leaf integrity may be challenging to breed for, but combining it with good agronomic practices, especially integrated pest management, and post-harvest handling, can help achieve this objective. The consistency of preferences among consumers and traders likely reflects their direct focus on product quality, whereas farmers operate in diverse production environments and livelihood strategies. As a result, farmers must balance agronomic and market-oriented traits, leading to a wider range of priorities. Previous studies support this, showing that farmers aim not only to maximize productivity but also to select varieties that meet consumer and buyer preferences [40,41]. An effective strategy for breeding programs would therefore be to consider farmer segmentation in TAV breeding while relying on the more stable preferences of consumers and traders to guide the selection of market-oriented traits.

For amaranth and jute mallow, farmers primarily valued disease resistance, color, multiple harvests (regenerative ability), and branching. Traits such as branching and multiple harvests directly influence yield and harvesting duration, while color is critical for marketability and trader or consumer acceptance. These preferences resemble the priority traits documented in previous studies in both West Africa and central Africa. For instance, Mitchodigni et al. [19] reported that late flowering, high branching, suitability for multiple harvesting, broad leaves, and green to dark green color were key traits farmers sought in amaranth and jute mallow. Similarly, Ayenan et al. [22] found that farmer-preferred traits in Benin included branching, marketability, late flowering, and cooking quality. Voss et al. [21] also noted that farmers’ preferences for amaranth varieties in Tanzania were driven primarily by plant survival, yield, leaf size, taste, and marketability.

This overlap among farmers, traders, and consumers’ priorities presents an opportunity for breeders to develop varieties that meet these common preferences [42]. Integrating these shared preferences into breeding objectives could accelerate adoption and strengthen the link between breeding programs, local markets, and consumer demand across TAV value chains.

Compared to other crops, our results show that okra farmers prioritize productivity-related agronomic traits over marketability traits. This focus on productivity likely reflects okra’s dual role as both a cash and subsistence crop [43]. Another reason farmers consider disease resistance important is that the crop is highly susceptible to pests and diseases, which can lead to total crop failure if not properly managed. Since 2022, an outbreak of jassids, a leafhopper, has affected okra in West Africa [44], prompting farmers to increase pesticide use. Additionally, viral diseases, especially begomoviruses, are common in okra [45]. From a breeding perspective, these findings suggest that developing okra varieties with improved yield stability, disease resistance, and longer fruiting periods could greatly enhance adoption among farmers. However, incorporating market-oriented traits like fruit color, size, and high viscosity into breeding goals remains essential to meet the expectations of traders and consumers, thereby strengthening the entire value chain. Accordingly, integrating both agronomic traits and market-based attributes remains a key challenge for breeding programs.

Our findings underscore the importance of tailoring TAV breeding to account for the diverse preferences associated with geographic location, gender, and age. Effective breeding programs must suit diverse contexts, and typically focus on low-input systems and farmers within defined geographic areas who share common interests in conserving and enhancing their genetic resources [46]. Regarding gender, both men and women participate in all activities along the amaranth, okra, and jute mallow value chains, from production to consumption, and display notable differences in their trait preferences, especially for amaranth and okra. Recognizing these differences, breeding programs must understand and address the needs and constraints of both men and women. A gender-sensitive breeding approach is recommended, rather than traditional breeding, which often overlooks varietal traits prioritized by women [47]. Such an approach has proven effective not only in promoting equity but also in producing more widely adopted and impactful crop varieties [48,49]. As Fischer et al. [50] highlighted, equity is a key prerequisite for sustainable vegetable farming.

Furthermore, this study revealed notable differences in trait preferences between younger and older farmers and consumers, especially for amaranth and jute mallow. Younger people tend to focus on traits related to productivity and marketability, while older people are more guided by tradition and cultural norms, which significantly influence their trait choices [51]. This aligns with Yila et al. [52], who found that within the same gender group, youth and older adults showed different preferences for production, market, and nutritional varietal traits. These findings indicate that TAV breeding programs should consider both age and gender segmentation to potentially improve the adoption and commercialization of enhanced varieties.

This study found no significant segmentation among TAV traders by country, gender, or age, indicating relative homogeneity in their trait preferences. This could be because traders often operate under similar market conditions, which causes their preferences for varietal traits to align. Such alignment likely results from practical factors like profitability, consistent supply, and marketability rather than socio-demographic differences. In other words, unlike farmers and consumers, traders’ varietal preferences may be more influenced by commercial considerations [53] than by cultural or demographic differences.

In summary, the findings of this study highlight that effective TAV breeding programs must balance market-oriented traits with locally relevant agronomic characteristics, tailoring strategies to the specific needs of diverse farmer communities. Incorporating market segmentation—including considerations of age, gender, and geographic context—could enhance the adoption and conservation of genetic resources. Meanwhile, consistent preferences among consumers and traders can guide the selection of traits with high market potential. This integrated approach has important implications for seed system design and value chain development, emphasizing the need for context-specific varieties, inclusive participation, and alignment between production realities and market demands. A key limitation of this study is the hypothetical nature of the gamified choice experiment, which may limit how directly the findings reflect actual choices under real-world constraints. Nevertheless, the study provides valuable insights for designing effective breeding programs to support the dissemination of improved TAV varieties in West Africa.

## 5. Conclusion

This study assesses the diverse trait preferences of farmers, traders, and consumers for amaranth, okra, and jute mallow varieties in Benin and Mali. Results from the Bradley–Terry model reveal distinct prioritization patterns that reflect region-, age-, and gender-specific needs. For consumers and traders, traits such as leaf integrity and taste in amaranth, viscosity in okra, and both leaf integrity and viscosity in jute mallow emerged as the most valued attributes. In contrast, traits like leaf size, fruit size, and freshness were less prioritized by these groups. Farmers, however, expressed preferences based on both agronomic importance and market value. They particularly valued disease resistance, plant regenerative capacity, harvest time, number of fruits per plant, and leaf and pod color across the three crops, with notable differences observed between countries, age groups, and genders. These findings emphasize the importance of local TAV breeding programs responsive to the diverse needs of farmers, trades and consumers. The alignment of breeding goals with their priorities could improve the adoption of improved TAV varieties and strengthen local value chains, contributing to food and nutritional security.

## Supporting information

S1 Table Bradley–Terry model results of farmers’ pairwise choice game for amaranth traits, with recursive partitioning (N = 189). (PDF)

S2 Table Bradley–Terry model results of farmers’ pairwise choice game for okra traits, with recursive partitioning (N = 189). (PDF)

S3 Table Bradley–Terry model results of farmers’ pairwise choice game for jute mallow traits, with recursive partitioning (N = 189). (PDF)

S4 Table Bradley–Terry model results of consumers’ pairwise choice game for amaranth traits, with recursive partitioning (N = 1336). (PDF)

S5 Table Bradley–Terry model results of consumers’ pairwise choice game for okra traits, with recursive partitioning (N = 1336). (PDF)

S6 Table Bradley–Terry model results of consumers’ pairwise choice game for jute mallow traits, with recursive partitioning (N = 1336). (PDF)
