# Supplementary material for: A gamified choice experiment of traditional African vegetable varieties in West Africa
Source: PLoS One. 2026 Mar 25;21(3):e0345915. doi: 10.1371/journal.pone.0345915 (PMC13016308; doi:10.1371/journal.pone.0345915)
Supplement: S1 Table — (PDF) [file pone.0345915.s001.pdf]

S1 Table. Bradley–Terry model results of farmers' pairwise choice game for amaranth traits, with recursive partitioning (N = 189)

**Traits**	**Normalized worth estimates**	**Standard error**	Z **value**	$p(> \|z\|)$		**Log-Likelihood**
Node 2						-901.4
Color	0.302	0.145	10.619	<0.001	***	
Leaf size	0.211	0.142	8.265	<0.001	***	
Multiple harvest	0.152	0.139	6.087	<0.001	***	
Flowering	0.064	0.141	-0.141	0.888		
Branching	0.145	0.141	5.704	<0.001	***	
Resistance to diseases	0.061	0.142	-0.496	0.620		
Drought tolerance	0.065					
Node 4						-132.8
Color	0.054	0.421	-4.602	<0.001	***	
Leaf size	0.077	0.430	-3.662	<0.001	***	
Multiple harvest	0.112	0.425	-2.836	0.005	**	
Flowering	0.031	0.501	-4.950	<0.001	***	
Branching	0.177	0.443	-1.678	0.093		
Resistance to diseases	0.177	0.443	-1.678	0.093		
Drought tolerance	0.372					
Node 5						-395.0
Color	0.124	0.214	-4.512	<0.001	***	
Leaf size	0.073	0.222	-6.735	<0.001	***	
Multiple harvest	0.049	0.229	-8.271	<0.001	***	
Flowering	0.031	0.247	-9.508	<0.001	***	
Branching	0.126	0.219	-4.360	<0.001	***	
Resistance to diseases	0.269	0.221	-0.881	0.378		
Drought tolerance	0.327					

*p < 0.05, **p < 0.01, ***p < 0.001. We used *drought tolerance* as the reference trait.
