# Supplementary material for: A gamified choice experiment of traditional African vegetable varieties in West Africa
Source: PLoS One. 2026 Mar 25;21(3):e0345915. doi: 10.1371/journal.pone.0345915 (PMC13016308; doi:10.1371/journal.pone.0345915)
Supplement: S2 Table — (PDF) [file pone.0345915.s002.pdf]

S2 Table. Bradley–Terry model results of farmers' pairwise choice game for okra traits, with recursive partitioning (N = 189)

**Traits**	**Normalized worth estimates**	**Standard error**	Z **value**	$p(> \|z\|)$		**Log-Likelihood**
Node 2						-1182.0
Color	0.130	0.128	13.697	<0.001	***	
Size	0.031	0.122	2.584	0.010	**	
Time to the first fructification	0.046	0.121	5.871	<0.001	***	
Harvesting duration	0.077	0.123	9.969	<0.001	***	
Number of fruits per plant	0.127	0.127	13.521	<0.001	***	
Resistance to diseases	0.567	0.159	20.258	<0.001	***	
Drought tolerance	0.023					
Node 4						-596.9
Color	0.068	0.153	-1.219	0.223		
Size	0.005	0.218	-12.908	<0.001	***	
Time to the first fructification	0.0158	0.173	-9.508	<0.001	***	
Harvesting duration	0.055	0.153	-2.645	0.008	**	
Number of fruits per plant	0.081	0.153	-0.077	0.939		
Resistance to diseases	0.693	0.221	9.631	<0.001	***	
Drought tolerance	0.082					
Node 5						-491.6
Color	0.095	0.178	1.848	0.065		
Size	0.033	0.187	-3.930	<0.001	***	
Time to the first fructification	0.047	0.181	-2.052	0.040	*	
Harvesting duration	0.097	0.178	1.935	0.053		
Number of fruits per plant	0.159	0.183	4.592	<0.001	***	
Resistance to diseases	0.500	0.220	9.036	<0.001	***	
Drought tolerance	0.069					

*p < 0.05, **p < 0.01, ***p < 0.001. We used *drought tolerance* as the reference trait.
