# Supplementary material for: A gamified choice experiment of traditional African vegetable varieties in West Africa
Source: PLoS One. 2026 Mar 25;21(3):e0345915. doi: 10.1371/journal.pone.0345915 (PMC13016308; doi:10.1371/journal.pone.0345915)
Supplement: S3 Table — (PDF) [file pone.0345915.s003.pdf]

S3 Table. Bradley–Terry model results of farmers' pairwise choice game for jute mallow traits, with recursive partitioning (N = 189)

**Traits**	**Normalized worth estimates**	**Standard error**	Z **value**	$p(>\|z\|)$		**Log-Likelihood**
Node 3						-864.0
Color	0.100	0.140	0.838	0.402		
Leaf size	0.154	0.140	3.964	<0.001	***	
Regeneration ability	0.153	0.140	3.897	<0.001	***	
Flowering	0.170	0.141	4.627	<0.001	***	
Branching	0.235	0.144	6.775	<0.001	***	
Resistance to diseases	0.100	0.140	0.838	0.402		
Tolerance to water stress	0.089					
Node 4						-703.5
Color	0.088	0.155	-3.771	<0.001	***	
Leaf size	0.115	0.153	-2.124	0.034	*	
Regeneration ability	0.094	0.154	-3.398	0.001	***	
Flowering	0.136	0.153	-0.990	0.322		
Branching	0.290	0.160	3.784	<0.001	***	
Resistance to diseases	0.119	0.153	-1.898	0.058		
Tolerance to water stress	0.159					
Node 5						-490.7
Color	0.079	0.198	-7.191	<0.001	***	
Leaf size	0.104	0.195	-5.891	<0.001	***	
Regeneration ability	0.052	0.206	-9.000	<0.001	***	
Flowering	0.068	0.201	-7.856	<0.001	***	
Branching	0.116	0.195	-5.338	<0.001	***	
Resistance to diseases	0.252	0.196	-1.359	0.174		
Tolerance to water stress	0.329					

*p < 0.05, **p < 0.01, ***p < 0.001. We used *tolerance to water stress* as the reference trait.
