# Supplementary material for: A gamified choice experiment of traditional African vegetable varieties in West Africa
Source: PLoS One. 2026 Mar 25;21(3):e0345915. doi: 10.1371/journal.pone.0345915 (PMC13016308; doi:10.1371/journal.pone.0345915)
Supplement: S4 Table — (PDF) [file pone.0345915.s004.pdf]

S4 Table. Bradley–Terry model results of consumers' pairwise choice game for amaranth traits, with recursive partitioning (N = 1336)

Traits	Normalized worth estimates	Standard error	Z value	$p(> \|z\|)$		Log-Likelihood
Node 3						-2927.0
Size	0.035	0.070	-22.420	<0.001	***	
Freshness	0.116	0.059	-6.464	<0.001	***	
Color	0.245	0.059	6.183	<0.001	***	
Taste	0.435	0.063	14.985	<0.001	***	
Physical integrity of leaves	0.170					
Node 4						-406.7
Size	0.068	0.174	-5.483	<0.001	***	
Freshness	0.166	0.161	-0.403	0.687		
Color	0.239	0.162	1.849	0.065		
Taste	0.350	0.167	4.074	<0.001	***	
Physical integrity of leaves	0.177					
Node 6						-3651.0
Size	0.047	0.064	-28.861	<0.001	***	
Freshness	0.185	0.054	-8.845	<0.001	***	
Color	0.191	0.054	-8.329	<0.001	***	
Taste	0.278	0.054	-1.327	0.185		
Physical integrity of leaves	0.299					
Node 7						-595.1
Size	0.040	0.157	-10.933	<0.001	***	
Freshness	0.166	0.131	-2.214	0.027	*	
Color	0.162	0.131	-2.407	0.016	*	
Taste	0.411	0.137	4.483	<0.001	***	
Physical integrity of leaves	0.222					

*$p < 0.05$, **$p < 0.01$, ***$p < 0.001$. We used *physical integrity of leaves* as the reference trait.
