# Supplementary material for: A gamified choice experiment of traditional African vegetable varieties in West Africa
Source: PLoS One. 2026 Mar 25;21(3):e0345915. doi: 10.1371/journal.pone.0345915 (PMC13016308; doi:10.1371/journal.pone.0345915)
Supplement: S5 Table — (PDF) [file pone.0345915.s005.pdf]

S5 Table. Bradley–Terry model results of consumers' pairwise choice game for okra traits, with recursive partitioning (N = 1336)

Traits	Normalized worth estimates	Standard error	Z value	$p(>\|z\|)$		Log-Likelihood
Node 3						-4340.0
Size	0.130	0.058	-15.832	<0.001	***	
Freshness	0.192	0.057	-9.266	<0.001	***	
Color	0.150	0.058	-13.451	<0.001	***	
Hardness	0.153	0.058	-13.092	<0.001	***	
Texture	0.048	0.065	-29.632	<0.001	***	
Viscosity	0.327					
Node 4						-1376.0
Size	0.115	0.107	-11.323	<0.001	***	
Freshness	0.177	0.106	-7.448	<0.001	***	
Color	0.127	0.107	-10.522	<0.001	***	
Hardness	0.137	0.106	-9.837	<0.001	***	
Texture	0.056	0.115	-16.914	<0.001	***	
Viscosity	0.389					
Node 6						-5614.0
Size	0.058	0.059	-36.96	<0.001	***	
Freshness	0.119	0.056	-25.90	<0.001	***	
Color	0.141	0.056	-23.05	<0.001	***	
Hardness	0.137	0.056	-23.52	<0.001	***	
Texture	0.040	0.061	-41.69	<0.001	***	
Viscosity	0.507					
Node 7						-1137.0
Size	0.074	0.123	-13.471	<0.001	***	
Freshness	0.138	0.117	-8.716	<0.001	***	
Color	0.171	0.116	-6.911	<0.001	***	
Hardness	0.169	0.116	-7.011	<0.001	***	
Texture	0.070	0.122	-13.813	<0.001	***	
Viscosity	0.380					

*$p < 0.05$, **$p < 0.01$, ***$p < 0.001$. We used *viscosity* as the reference trait.
