# Supplementary material for: A gamified choice experiment of traditional African vegetable varieties in West Africa
Source: PLoS One. 2026 Mar 25;21(3):e0345915. doi: 10.1371/journal.pone.0345915 (PMC13016308; doi:10.1371/journal.pone.0345915)
Supplement: S6 Table — (PDF) [file pone.0345915.s006.pdf]

S6 Table. Bradley–Terry model results of consumers' pairwise choice game for jute mallow traits, with recursive partitioning (N = 1336)

**Traits**	**Normalized worth estimates**	**Standard error**	Z **value**	$p(>\|z\|)$		**Log-Likelihood**
Node 3						-937.6
Size	0.043	0.129	-16.452	<0.001	***	
Freshness	0.166	0.109	-7.091	<0.001	***	
Color	0.150	0.109	-7.950	<0.001	***	
Viscosity	0.282	0.108	-2.254	0.024	*	
Physical integrity of leaves	0.359					
Node 4						-3096.0
Size	0.033	0.074	-31.765	<0.001	***	
Freshness	0.126	0.060	-16.677	<0.001	***	
Color	0.186	0.059	-10.476	<0.001	***	
Viscosity	0.311	0.059	-1.729	0.084		
Physical integrity of leaves	0.344					
Node 5						-4262
Size	0.051	0.058	-32.604	<0.001	***	
Freshness	0.132	0.052	-18.150	<0.001	***	
Color	0.134	0.052	-17.930	<0.001	***	
Viscosity	0.345	0.052	0.385	0.700		
Physical integrity of leaves	0.338					

*p < 0.05, **p < 0.01, ***p < 0.001. We used *physical integrity of leaves* as the reference trait.
